# Occult Thyroid Cancer in Autoimmune Thyroiditis: Lymph Node Ultrasound as the Sole Diagnostic Indicator of Malignancy in a Pediatric Case of Papillary Thyroid Carcinoma

**DOI:** 10.3390/children12020194

**Published:** 2025-02-06

**Authors:** Maria Szwarkowska, Konrad Kaleta, Aleksandra Jurek, Monika Kujdowicz, Anna Taczanowska-Niemczuk, Aleksandra Kiszka-Wiłkojć, Marcin Maślanka, Wojciech Górecki, Jerzy Starzyk, Dominika Januś

**Affiliations:** 1 Students’ Scientific Group of Pediatric Auxology, Faculty of Medicine, Jagiellonian University Medical College, University Children’s Hospital in Krakow, Wielicka 265, 30-663 Krakow, Poland; maria.szwarkowska@student.uj.edu.pl (M.S.); konrad.kaleta@student.uj.edu.pl (K.K.); aleksandra.jurek@student.uj.edu.pl (A.J.); 2 Department of Pathomorphology, Jagiellonian University Medical College, 31-121 Krakow, Poland; monika.kujdowicz@uj.edu.pl; 3 Department of Pathology, University Children Hospital in Krakow, 30-663 Krakow, Poland; 4 Department of Pediatric Surgery, Institute of Pediatrics, Jagiellonian University Medical College, 31-121 Krakow, Poland; anna.taczanowska-niemczuk@uj.edu.pl (A.T.-N.); aleksandra.kiszka-wilkojc@uj.edu.pl (A.K.-W.); marcin.1.maslanka@uj.edu.pl (M.M.); wojciech.gorecki@uj.edu.pl (W.G.); 5 Department of Pediatric and Adolescent Endocrinology, Chair of Pediatrics, Institute of Pediatrics, Jagiellonian University Medical College, 32-121 Krakow, Poland; jerzy.starzyk@uj.edu.pl; 6 Department of Pediatric and Adolescent Endocrinology, University Children Hospital in Krakow, 30-663 Krakow, Poland

**Keywords:** pediatric papillary thyroid carcinoma, lymph node metastasis, psammoma bodies, autoimmune thyroiditis, lymph node ultrasound

## Abstract

Background: Autoimmune thyroiditis (AIT) is a common thyroid disorder in children, linked to an increased risk of papillary thyroid carcinoma (PTC). Characteristic ultrasonographic features of AIT can obscure PTC, delaying diagnosis. Case Presentation: An 11-year-old girl with a two-year history of AIT presented with persistently elevated thyroid-stimulating hormone (TSH) levels despite levothyroxine therapy. Examination revealed a firm, slightly enlarged right thyroid lobe. Serial thyroid ultrasounds showed typical AIT features, with no apparent tumor. However, a cervical lymph node ultrasound detected a suspicious lymph node with pathological vascularization. Fine-needle aspiration suggested possible PTC metastasis. The patient underwent total thyroidectomy with central and right lateral neck dissection. Histopathology confirmed multifocal PTC with cervical lymph node metastases (pT3aN1bM0). Postoperative radioactive iodine therapy resulted in undetectable thyroglobulin levels, indicating a biochemical response. Conclusions: Children with AIT may harbor occult PTC even without thyroid gland abnormalities suggestive of malignancy. Comprehensive ultrasound evaluation, including cervical lymph nodes, is vital for early detection and timely treatment.

## 1. Introduction

Papillary thyroid carcinoma (PTC) constitutes 80–90% of all thyroid cancers diagnosed in children [1,2,3]. Among pediatric patients with autoimmune thyroiditis (AIT), the prevalence of PTC ranges from 0.67% to 3%, a rate significantly higher than the baseline risk of approximately 0.02% in the general pediatric population [4]. From a surgical perspective, AIT is frequently observed in pediatric patients with PTC, with reported rates of coexistence reaching up to 50% in surgical series [4,5].

It remains unclear whether the elevated prevalence of PTC in patients with AIT reflects a genuine increase in risk or is primarily attributable to enhanced detection through routine ultrasound screening. Autoimmune thyroiditis, the most common autoimmune thyroid disorder, is widely recognized as an independent risk factor for PTC [6,7,8,9]. Moreover, recent studies have documented a rising prevalence of thyroid autoimmunity in at-risk pediatric populations, underscoring the need to better understand the relationship between AIT and PTC [10].

The reported prevalence of thyroid cancer associated with AIT varies significantly across healthcare centers, influenced by differences in diagnostic protocols and resources. At our tertiary thyroid care center, the availability of in-office ultrasound equipment facilitates the early detection of suspicious changes in patients with AIT. Nevertheless, we are observing an increasing number of referrals for patients presenting with large, multi-centimeter PTC lesions.

In experienced hands, early-stage thyroid cancer can often be detected in patients with AIT. However, interpreting ultrasound findings in pediatric cases remains challenging, particularly in instances where small, scattered cancer foci do not manifest as distinct lesions. Such findings risk being misclassified as inflammatory changes. Of concern, there is a growing trend of discontinuing ultrasound surveillance after a “reassuring” diagnosis of AIT, despite established guidelines recommending annual ultrasound evaluations for this patient population [1].

Advances in diagnostic evaluation, particularly neck ultrasonography (US) and fine-needle aspiration biopsy (FNAB), have significantly enhanced the detection of microcarcinomas, contributing to the rising number of reported cases over the past decade [3,11,12].

The ultrasonographic features characteristic of PTC include solid hypoechoic nodules with irregular margins and shape, extrathyroidal extension, a taller-than-wide configuration, microcalcifications (punctate echogenic foci), and cervical lymphadenopathy [1]. In pediatric patients, the primary presentation of PTC is typically a thyroid nodule. However, it may also present as cervical lymphadenopathy, with or without a palpable thyroid nodule, or as an incidental lesion identified during neck imaging or surgery performed for unrelated reasons [1].

Autoimmune thyroiditis and papillary thyroid carcinoma frequently exhibit overlapping clinical features, including goiters and both functional and structural thyroid abnormalities, complicating the distinction between benign and malignant conditions. This challenge is particularly pronounced in early-stage PTC or specific variants seen in pediatric patients [1,13]. A notable subtype, pediatric diffuse sclerosing PTC, is characterized by widespread infiltration leading to enlargement of the affected thyroid lobe or the entire gland, often accompanied by palpable cervical lymphadenopathy [1,9]. This variant is commonly associated with microcalcifications, necessitating fine-needle aspiration biopsy for definitive diagnosis [1,13].

Classic ultrasound (US) criteria for differentiating benign from malignant lymph nodes in children are similar to those used in adults [1]. B-scan criteria for benign lymph nodes include small size, oval shape, the presence of a hilum, moderate or low echogenicity, and sharply defined margins. Soft tissue edema may also be observed. Doppler ultrasound findings indicative of benign lymph nodes include absent vascular flow, centrally located vessels, a single vascular pattern, and low impedance values [14].

B-scan criteria for malignant lymph nodes include large size, rounded shape, absence of a hilum, marked hypoechogenicity, irregular, blurred, angular, or invasive margins, and structural changes such as focal cortical nodules, intranodal necrosis, reticulation, calcification, or matting [14]. Soft tissue edema is typically absent. Doppler ultrasound findings consistent with malignancy include the presence of vascular flow, peripherally located vessels, multiple vascular pedicles, a disorganized (chaotic) vascular pattern, and high impedance values [14]. Knowledge of these differences, coupled with improved access to high-resolution ultrasound equipment, facilitates the timely detection of suspected malignancies in at-risk patient groups.

Here, we report the case of an 11-year-old patient with a history of AIT, where cervical lymph node ultrasound was instrumental in raising suspicion of PTC in the context of an inflamed thyroid gland.

## 2. Methods

The patient’s TSH, fT3, and fT4 levels were measured using chemiluminescence analysis (Advia Centaur XPT; Siemens, Berlin, Germany). Thyroperoxidase antibodies (TPOAb) and thyroglobulin antibodies (TgAb) were evaluated using a radioimmunoassay method (BRAHMS, ThermoFisher Scientific, Waltham, MA, USA) at the Biochemistry Department of the University Children’s Hospital in Krakow, Poland. Ultrasound imaging was carried out by D.J. and A.K.-W., who hold certifications in pediatric ultrasonography, utilizing the Samsung HS40 system (Samsung Healthcare, Suwon, Republic of Korea) with linear probes. Fine-needle aspiration biopsies were assessed according to the Bethesda classification, as previously outlined [11]. A total thyroidectomy with lymph node dissection and subsequent histopathological evaluation was conducted by the Departments of Pediatric Surgery and Pathology. Hematoxylin and eosin (HE)-stained tissue sections (prepared at a thickness of 3.5 µm following deparaffinization) were scanned using the NanoZoomer SQ system (Hamamatsu Photonics, Hamamatsu, Japan). The images were obtained from these digital scans.

This study was approved by the relevant institutional review board (The Bioethics Committee of the Jagiellonian University opinion number:118.0043.1.103.2024 issued on 19 April 2024).

## 3. Case Report

An 11-year-old female patient with a two-year history of autoimmune thyroiditis presented to the outpatient thyroid clinic with complaints of difficulty waking in the mornings, persistent hair loss, sensations of heat, and mild weight gain over the past few months. Her family history indicated maternal hypothyroidism and vitiligo, and she had a paternal grandmother who had undergone a thyroidectomy for thyroid follicular nodular disease.

Initial laboratory investigations from her past medical records showed significantly elevated thyroid-stimulating hormone (TSH) levels at 19.53 μIU/mL (reference range: 0.3–4.0 μIU/mL), with normal free triiodothyronine (fT3) at 5.59 pmol/L (4.0–7.8 pmol/L) and free thyroxine (fT4) at 12.09 pmol/L (10.0–25.0 pmol/L). Thyroid autoantibodies were elevated, with TPOAb at 36 IU/mL (reference <20 IU/mL) and TgAb at 47 IU/mL (reference <30 IU/mL). She was started on levothyroxine therapy (50 μg daily).

An initial thyroid ultrasound performed during an earlier consultation demonstrated a heterogeneous, hypoechoic thyroid gland consistent with autoimmune thyroiditis. The right thyroid lobe measured 1.3 × 1.32 × 3.6 cm, the left lobe measured 1.58 × 1.26 × 3.12 cm, and the isthmus was 0.26 cm thick. No cystic or solid nodules were identified at that time.

At the follow-up visit, despite ongoing levothyroxine therapy, her TSH levels remained elevated at 13.17 μIU/mL, likely due to an insufficient dosage adjustment by the managing physician. Additionally, there was a marked increase in thyroid autoantibodies over time, with TPOAb rising from 36 IU/mL to 562.6 IU/mL and TgAb increasing from 47 IU/mL to >4000 IU/mL. The patient’s mother reported noticeable thyroid enlargement, prompting a repeat thyroid ultrasound.

Physical examination revealed a mildly enlarged thyroid gland with a firm consistency, particularly pronounced in the right lobe. Palpation of the neck also identified a firm lesion measuring 1 × 1.5 cm on the right side of the thyroid, located in the cervical level III.

**Figure 1:**
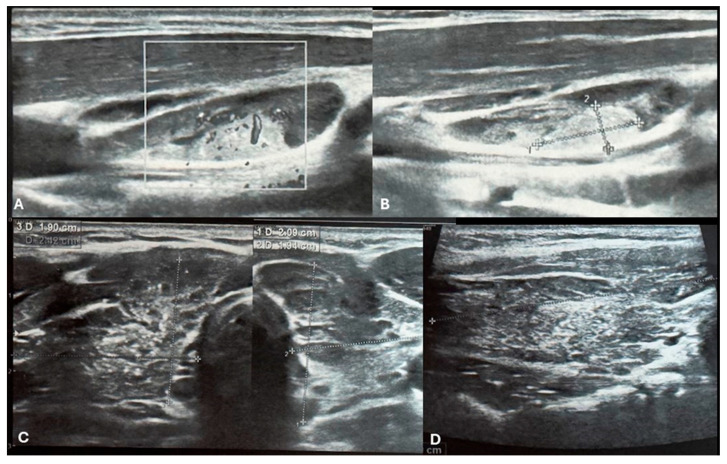
Ultrasound imaging of the thyroid and neck region. (**A**,**B**) Ultrasound images depict a vascularized hyperechoic lesion measuring 1.01 × 0.45 cm located within a lymph node in the cervical level III. This finding raises suspicion for malignancy due to pathological vascularization and its proximity to the right thyroid lobe. (**C**) Transversal images of right and left lobe showing remodeled thyroid gland as a result of autoimmune thyroiditis. (**D**) Longitudinal image of the right lobe. The thyroid parenchyma demonstrates characteristic heterogeneity, consistent with the chronic inflammatory changes seen in AIT. Additionally, multiple small microcalcifications are visible throughout the right thyroid lobe (**C**,**D**).

Repeat ultrasonography revealed multiple small microcalcifications scattered throughout the right thyroid lobe. A firm, enlarged neck nodule, palpable laterally to the right thyroid lobe, corresponded to a clearly suspicious lymph node on ultrasound located in level III on the right side. This lymph node appeared heterogeneous, contained microcalcifications, measured 20 × 10 mm, and exhibited structural features resembling infiltration of the right thyroid lobe [Figure 1A–D].

### 3.1. Fine-Needle Aspiration Biopsy and Surgical Plan

Fine-needle aspiration biopsy of the right thyroid lobe (diffuse infiltration of the entire right lobe without focal changes on US) was performed, revealing cytological findings of dispersed groups and sheets of thyrocytes with nuclear enlargement, clearing, occasional intranuclear grooves, and focal oxyphilic metaplasia. The background contained numerous lymphocytes, scattered multinucleated giant cells, colloid, and blood. These findings were classified as Bethesda category III (atypia of undetermined significance, AUS) with concurrent features of chronic lymphocytic thyroiditis. Fine-needle aspiration biopsy of the suspicious lymph node was initially deferred due to the patient’s non-cooperation during the procedure.

**Figure 2:**
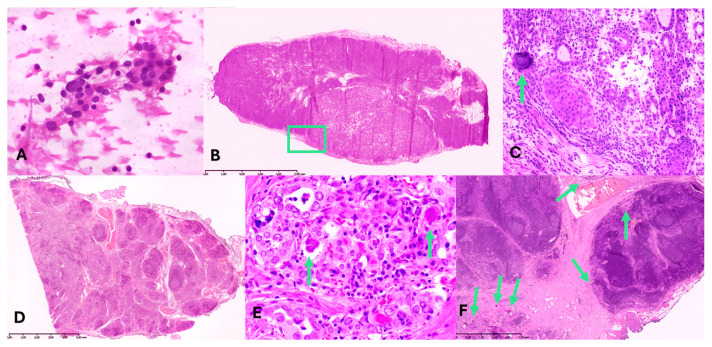
Morphologic findings. (**A**) Slide presenting cytological result from FNAB of the right thyroid lobe; (**B**)—intraoperative examination revealing lymph node metastasis; (**C**) Enlarged part of B containing psammoma body [green arrow] in metastasis; (**D**) Autoimmune thyroiditis’s-related changes seen in the left lobe of the thyroid gland; (**E**) Microfoci of carcinoma cells in the right thyroid lobe; (**F**) Fibrotic area with psammoma bodies in thyroid gland [down left], lymph node without metastasis [right], and parathyroid fat tissue [over the vessel].

Two weeks later, under sedation, FNAB was repeated, targeting a level III lymph node on the right side and another area of the infiltration in the right lobe. The results again indicated Bethesda III for the thyroid lesion [Figure 2A]. The lymph node aspirate revealed the presence of thyrocytes, suggestive of possible metastatic involvement. An assessment of the thyroglobulin from the fine-needle aspiration washouts was not performed as this is not included in the current Polish guidelines for the evaluation of differentiated thyroid carcinoma in children [1].

Preoperative diagnostics did not conclusively confirm malignancy during the FNAB. On a follow-up ultrasound performed prior to surgery, apart from the suspicious lymph node in level III, several small lymph nodes with microcalcifications were visualized in the lateral compartment at the boundary of levels II and IV. In level VI, a suspicious lymph node was identified below the lower pole of the right thyroid lobe. This node was round, measured 7 × 8 mm, and was deeply hypoechoic. Nearby, several other lymph nodes were observed adjacent to the isthmus and below the lower pole of the left thyroid lobe, demonstrating reactive features consistent with autoimmune thyroiditis.

Given the strong suspicion of thyroid cancer with metastases in the central compartment (level VI) and the right lateral compartment (levels III, as well as portions of levels II and IV) based on preoperative ultrasound findings, we adhered to the Polish Recommendations [1]. A central compartment lymphadenectomy (level VI) is recommended in pediatric patients diagnosed with thyroid cancer, regardless of ultrasound findings [1]. In a more limited approach, an ipsilateral central compartment lymphadenectomy (restricted to the side of the tumor/focal lesion) may also be performed in cases classified as Bethesda III or IV, depending on the ultrasound findings of the lesion and lymph nodes. Lateral neck lymphadenectomy (levels II–V) is performed only when metastatic involvement of the lateral lymph nodes has been confirmed through preoperative FNAB or intraoperative histopathological examination.

Based on the findings, a minimal intervention approach to surgical treatment was planned for our patient. Specifically, the procedure included a right lobectomy with isthmectomy, ipsilateral central compartment lymphadenectomy, and surgical biopsy (the complete removal of suspicious lymph nodes identified on ultrasound) of the level III lymph nodes on the right side. The option to extend the surgery was reserved, depending on the confirmation of malignancy through intraoperative histopathological examination of the level III lymph node. To ensure the integrity of the recurrent laryngeal nerve (RLN) and minimize the risk of injury, intraoperative RLN monitoring was employed.

### 3.2. Intraoperative Findings and Surgical Adjustments

Intraoperative frozen sections of the right lateral lymph node confirmed metastatic thyroid cancer in the level III lymph node on the right, leading to an agreed extension of the surgical plan [Figure 2B,C]. The final procedure included total thyroidectomy with central compartment lymphadenectomy, a modified right lateral neck dissection (levels II–V), and surgical biopsy of left lateral compartment level III lymph nodes.

Intraoperative examination also revealed small metastatic foci in level VI lymph nodes, fragments of which were submitted for histopathological assessment to differentiate between parathyroid tissue and lymph nodes.

The results of the intraoperative examination were relayed to the operating room via telephone, confirming malignancy with a clear diagnosis of “cancer.” However, the written report later expressed uncertainty, citing a lack of a dominant focal lesion in the right lobe and the presence of dispersed small cancer foci. These findings were ultimately confirmed by the National Institute of Oncology (NIO), consistent with preoperative ultrasound findings.

### 3.3. Histopathological Findings

Permanent histopathological analysis of the thyroid revealed extensive chronic lymphocytic thyroiditis (autoimmune thyroiditis), characterized by dense lymphocytic infiltration forming lymphoid follicles with germinal centers, prominent oxyphilic (Hürthle cell) metaplasia, and focal fibrosis. Numerous psammoma bodies were identified in the right lobe, predominantly in fibrotic areas adjacent to lymphatic vessels and lymph nodes. Minute foci (0.25–0.5 mm) of atypical follicular epithelial cells were noted, demonstrating nuclear features characteristic of papillary thyroid carcinoma, including nuclear enlargement, clearing, grooves, and occasional intranuclear inclusions [Figure 2D–F]. These findings confirmed the diagnosis of PTC, despite the absence of a dominant tumor mass. The maximum dimension of the affected lobe (4.5 cm) was recorded as the size of the primary tumor.

Immunohistochemical staining for cytokeratin 19 (CK19) confirmed the presence of PTC in atypical follicular epithelial cells and metastatic lymph nodes. Additionally, in the central neck lymph nodes (level VI), psammoma bodies were identified in 3 of the 27 nodes without overt metastatic carcinoma and microscopic foci of PTC metastases were also identified intraoperatively in two lymph nodes adjacent to the left thyroid lobe. In the right lateral lymph nodes (levels II–V), 5 out of 28 nodes were positive for metastases, with the largest metastasis measuring 11 mm, confined to the lymph nodes. Psammoma bodies were noted in two additional lymph nodes, while one lymph node contained ectopic thyroid tissue. In the left lateral lymph nodes (level III, surgical biopsy), 0 out of 4 nodes were positive for metastases.

The final pathological diagnosis was multifocal papillary thyroid carcinoma with diffuse involvement of the right thyroid lobe, scattered minute foci of carcinoma, psammoma bodies throughout the lobe, and metastatic involvement of the central (level VI) and right lateral neck lymph nodes (levels II–V). The diffuse sclerosing variant of PTC (dsvPTC) was identified. The tumor was staged as pT3aN1bM0 according to the AJCC 8th edition staging system [15].

### 3.4. Postoperative Course

The patient tolerated the surgery well, with no immediate complications. Her vocal cord function was intact, and her calcium levels remained within normal limits. Prophylactic calcium and vitamin D supplementation were initiated. The patient was discharged in a stable condition on suppressive levothyroxine therapy (Euthyrox 150 μg daily).

An intermediate risk of cancer (clinical feature N1, >5 lymph nodes with metastases, lymphovascular invasion) qualified her for iodine treatment according to national recommendations [1]. All details, warnings, and precautions related to iodine therapy remained under the purview of the nuclear medicine center. The patient and her family, after being informed about the potential side effects of radioiodine therapy (such as sialadenitis, fertility disorders, and the possibility of secondary malignancy), provided written consent for the treatment [1,16].

Four months postoperatively, the patient underwent radioactive iodine (RAI) ablation therapy with 100 mCi of I-131 following recombinant human TSH (rhTSH) stimulation. A post-therapy whole-body scan revealed no abnormal iodine uptake, indicating the absence of residual or metastatic iodine-avid disease. Serum thyroglobulin levels under rhTSH stimulation were undetectable (<0.04 ng/mL), with negative anti-thyroglobulin antibodies, reflecting an excellent biochemical response. Suppressive levothyroxine therapy was continued, targeting a TSH level of 0.1–0.4 μIU/mL.

## 4. Discussion

In this report, we present the case of a child with autoimmune thyroiditis complicated by papillary thyroid carcinoma. This case underscores the possibility of underlying PTC in children with AIT, even in the absence of detectable thyroid nodules or typical malignant lesions on an ultrasound. Additionally, we detail the entire diagnostic and therapeutic process, emphasizing adherence to the latest national recommendations from 2024. We also highlight that the extent of surgery was modified based on intraoperative histopathological findings, underscoring the critical role of an experienced pathologist in the dynamic decision-making process.

Papillary thyroid carcinoma, originating from the follicular cells of the thyroid gland, affects both adults and children, with a notable female predominance [4,17,18]. In pediatric cases, PTC often presents with distinct clinical and pathological features compared to its adult counterpart. Pediatric PTC is more likely to be multifocal and involve the entire gland. Tumors in children are generally larger at the time of diagnosis and exhibit a higher propensity for extrathyroidal extension, lymph node metastases, and recurrence [19,20,21]. Tumor size and metastatic patterns differ among pediatric age groups; children under 14 years are more likely to present with larger tumors and central cervical lymph node involvement [20]. Despite these aggressive features, pediatric PTC has an excellent prognosis, with 5-year survival rates of approximately 98% in children aged 0–14 years and 99% in adolescents aged 15–19 years [18]. Children have a longer life expectancy than adults, making it essential to carefully consider the long-term consequences of thyroid cancer treatment in pediatric patients. Radioiodine (RAI) therapy carries a risk of complications, including dysfunction of the salivary and lacrimal glands (the most common adverse effects), reductions in male and female fertility, bone marrow suppression, and an increased risk of secondary cancers [1,16]. Therefore, earlier detection of thyroid cancer offers hope for avoiding the need for RAI or other adjuvant therapies, thereby reducing the potential for long-term complications.

At our center, we currently apply the 2023 Bethesda system for thyroid cytology, which includes the following categories: I—Nondiagnostic; II—Benign; III—Atypia of Undetermined Significance (AUS; previously categorized as FLUS, Follicular Lesion of Undetermined Significance); IV—Follicular Neoplasm (FN; formerly SFN, Suspicion of Follicular Neoplasm); V—Suspicious for Malignancy; and VI—Malignant [22]. The risk of malignancy is notably higher in children than in adults, with this variability influenced by factors such as biopsy technique and the expertise of the evaluator. Consequently, different centers may report diverse data.

In pediatric patients, the risk of malignancy (ROM) for thyroid nodules is reported to be 29.6% for Bethesda category III, 42.3% for category IV, and 90.8% for category V [1]. However, it is worth noting that a study by Ronen et al., which included both children and adults, reported the risk of malignancy for Bethesda category IV as being high as 50% [23].

At our center, any biopsy result that could potentially lead to surgical treatment undergoes evaluation by two pathologists to ensure accuracy. Nevertheless, a biopsy result alone cannot, and should not, serve as the sole basis for determining the treatment approach. Treatment qualification must also consider ultrasound findings and the broader clinical context, including factors such as prior radiation exposure, the presence of specific genetic syndromes, and other relevant considerations.

Currently, there are few additional recommended methods to enhance the accuracy of fine-needle aspiration biopsy in pediatric patients. The assessment of thyroglobulin in fine-needle aspiration washouts is not advised as part of standard diagnostic protocols and is not included in the current Polish guidelines for the evaluation of differentiated thyroid carcinoma in children [1]. Consequently, this method is not utilized at our center. However, in equivocal cases, it may be considered as a supplementary diagnostic tool, particularly for assessing nodal lesions. Nonetheless, its diagnostic utility may be limited, as histopathological studies have documented the presence of benign thyroid follicular inclusions (based on our unpublished observations).

Similarly, genetic testing does not currently have an established role in pediatric patients. Its results do not provide sufficient guidance for determining the scope of surgical treatment in children, as specified in the Polish Recommendations [1]. Moreover, there are no current guidelines supporting routine molecular testing in this population [1].

Postoperative diagnosis of thyroid cancer for nodules classified as Bethesda categories III and IV is more frequent in children compared to adults. As a result, surgical intervention is strongly advised for pediatric patients with indeterminate cytology in these categories. According to the 2024 Polish Guidelines on Papillary Thyroid Carcinoma in children, the preferred surgical approach is lobectomy, including removal of the isthmus, rather than a repeated fine-needle aspiration biopsy [1]. Research by Kujdowicz et al. indicates that the sensitivity of FNAB in detecting non-benign neoplasms across Bethesda categories III to VI is approximately 86% for both autoimmune thyroiditis and non-AIT patients [11]. However, for papillary thyroid carcinoma, its sensitivity in categories V and VI is significantly reduced in patients with AIT, decreasing from 86% in non-AIT patients to 61.5% in AIT cases [11]. These findings underscore the importance of considering surgical treatment in pediatric patients with Bethesda III–VI cytology, especially in the presence of AIT, where its diagnostic accuracy is compromised.

Intraoperative examination may assist in determining the extent of lymph node dissection and differentiating between parathyroid tissue and lymph nodes. However, it is not routinely recommended for diagnosing thyroid nodules, particularly follicular lesions or small tumors [1]. Nonetheless, it may be beneficial in assessing suspicious lymph nodes, as illustrated in this case.

Although there is a growing trend to reduce the extent of surgical procedures, as highlighted in the recent study by Ji et al., which reported that a lobectomy does not adversely affect prognosis in cases of unilateral TNM T1 and T2 papillary thyroid carcinoma with unilateral lymph node metastasis, this study was limited to adults [24].

The situation is notably different in pediatric patients, where papillary thyroid carcinoma (PTC) is frequently bilateral and multifocal [1,11,13,25]. According to the Polish Recommendations, the diagnosis of thyroid cancer in a child necessitates total thyroidectomy [1]. Thyroid cancer with evident nodal metastases is classified as at least intermediate risk, requiring iodine therapy, which in turn mandates a prior total thyroidectomy [1]. In the case of this particular patient, leaving the left thyroid lobe intact and foregoing iodine therapy would have resulted in the retention of two metastatic lymph nodes located in the central compartment on the left side, adjacent to the left lobe of the thyroid, which would have significantly increased the risk of recurrence.

As presented in this report, patients and their guardians are always provided with written information regarding the risks and potential need for staged thyroidectomy. This includes the possibility of signal loss from the recurrent laryngeal nerve (RLN) on the initially operated side, as well as the potential need to extend the surgical treatment following an initial procedure that was less extensive than total thyroidectomy [1,26]. Written consent is obtained from guardians and from patients aged 16 years or older. The scope of information provided to younger patients is tailored to their age and agreed upon with their legal guardians.

Studies highlight notable differences between PTC in children [≤15 years] and adolescents [>15 years], particularly in clinical presentation and preoperative ultrasound [US] features [27,28]. Papillary thyroid carcinoma in younger children is generally more aggressive, with a higher likelihood of regional lymph node metastases, extrathyroidal invasion, and pulmonary metastases. These cases are also associated with higher pathological tumor–node–metastasis [pTNM] staging and a greater prevalence of microcalcifications within nodules, as seen on ultrasound [28]. Pediatric thyroid cancer should be suspected in the presence of a solid, rapidly growing thyroid nodule with calcifications. Additionally, cervical lymph node metastases should be strongly considered in patients with a PTC diagnosis [27].

A study comparing the US features of pediatric PTC patients with and without AIT found that most patients displayed typical features of PTC, including irregular shape and margins, solid nodules, hypoechogenicity, punctate echogenic foci (microcalcifications), and lymph node metastasis. Papillary thyroid carcinoma was also characterized by a wider-than-tall shape and predominantly intranodular vascularity, as opposed to peripheral vascularity [13,28,29].

Punctate echogenic foci were significantly more frequent in PTC patients with AIT compared to those without AIT [28].

Microscopically, PTC commonly displays fibrovascular papillae surrounded by one or more layers of cells with oval, crowded nuclei described as clear, empty, or “Orphan Annie-eyed” nuclei [30,31]. Psammoma bodies, which represent calcified remnants of necrotic papillae, are typically located within the tumor stroma or lymphatic vessels at the cores of papillae [31]. Several histologic subtypes of PTC have been identified apart from the classic variant, including the “high-risk” tall cell variant, follicular variant, and solid/trabecular variant, as well as the diffuse sclerosing variant [4,30]. The diffuse sclerosing variant is distinguished by the widespread involvement of one or both thyroid lobes, prominent squamous metaplasia, a high density of psammoma bodies, significant interstitial fibrosis, and marked lymphocytic infiltration with germinal center formation [30]. In pediatric PTC, the histologic features differ from those in adults, including a higher prevalence of psammoma bodies and rounded, non-overlapping nuclei [4]. The dsvPTC subtype may not form a distinct gross tumor but presents with diffuse glandular enlargement, squamous metaplasia, and psammoma bodies, with increased vascularity and microcalcifications frequently observed in pediatric cases [28,32,33,34]. In some cases, the presence of microcalcifications on thyroid imaging may be the only distinguishing feature between autoimmune thyroiditis and papillary thyroid carcinoma, as observed in the present case and previous reports [32,33,34,35,36,37]. The similar echo structure of dsvPTC and benign conditions, such as AIT, likely contributes to the delayed diagnosis [36,37].

The ultrasonographic characteristics of thyroid cancer in patients with AIT remain poorly documented [1,11,13,25]. However, cases like this highlight the critical importance of performing detailed ultrasound evaluations, including thorough examination of cervical lymph nodes, to identify potential abnormalities and improve diagnostic accuracy.

The pathogenesis linking autoimmune thyroiditis and thyroid cancer remains poorly understood. One hypothesis suggests that AIT, as a chronic inflammatory condition, induces structural damage to the thyroid gland, creating a microenvironment conducive to carcinogenesis. Another hypothesis posits that organ-specific regulatory T-cell dysfunction in AIT patients impairs local immune surveillance, facilitating the development of thyroid cancer [5]. Molecular studies have implicated genes such as p53, BCL-2, and RET in thyroid cancer pathogenesis, offering further insights into its development [35]. Autoimmune thyroiditis is often characterized by dense lymphocytic infiltration and lymphoid follicle formation, where chronic antigenic stimulation may drive neoplastic hyperplasia of thyroid follicles, ultimately leading to malignant transformation. Additionally, high iodine intake, which increases AIT prevalence, may exacerbate thyroid epithelial cell damage and immune dysregulation, further promoting carcinogenesis [35].

In the present case, the patient exhibited persistently elevated thyroid-stimulating hormone levels despite levothyroxine therapy, likely due to an insufficient dosage adjustment by the managing physician. This was accompanied by progressively rising levels of TPOAb and TgAb. Interestingly, Min et al. identified a significant association between elevated serum TgAb levels and central lymph node metastasis (LNM) in patients with PTC and AIT, suggesting TgAb as a potential biomarker for LNM in this population [38]. Furthermore, Min et al. demonstrated in adults that a TgAb/TPOAb ratio exceeding two was strongly correlated with more extensive disease [38]. Similarly, Xu et al. reported that larger PTC lesions and LNM were significantly associated with elevated TgAb levels, reinforcing its value as a biomarker for disease severity [39]. Given the limited data in pediatric series, multicenter research is crucial to validate these findings and better understand their clinical implications in children [25].

## 5. Conclusions

Children with AIT may harbor occult PTC even in the absence of thyroid abnormalities suggestive of malignancy. A comprehensive ultrasound evaluation, including detailed assessment of cervical lymph nodes, is essential for early detection and timely treatment.

## Data Availability

The raw data supporting the conclusions of this article will be made available by the authors on request.
